# Conformal Flattening for Deformed Information Geometries on the Probability Simplex †

**DOI:** 10.3390/e20030186

**Published:** 2018-03-10

**Authors:** Atsumi Ohara

**Affiliations:** Department of Electrical and Electronics, University of Fukui, Bunkyo, Fukui 910-8507, Japan; ohara@fuee.u-fukui.ac.jp

**Keywords:** representing functions, affine immersion, nonextensive statistical physics, invariance, dually flat structure, Legendre conjugate, gradient flow

## Abstract

Recent progress of theories and applications regarding statistical models with generalized exponential functions in statistical science is giving an impact on the movement to deform the standard structure of information geometry. For this purpose, various representing functions are playing central roles. In this paper, we consider two important notions in information geometry, i.e., invariance and dual flatness, from a viewpoint of representing functions. We first characterize a pair of representing functions that realizes the invariant geometry by solving a system of ordinary differential equations. Next, by proposing a new transformation technique, i.e., conformal flattening, we construct dually flat geometries from a certain class of non-flat geometries. Finally, we apply the results to demonstrate several properties of gradient flows on the probability simplex.

## 1. Introduction

The theory of information geometry has elucidated abundant geometric properties equipped with a Riemannian metric and mutually dual affine connections. When it is applied to the study of statistical models described by the exponential family, the logarithmic function plays a significant role in giving the standard information geometric structure to the models [1,2].

Inspired by the recent progress of several areas in statistical physics and mathematical statistics [3,4,5,6,7,8,9,10] which have exploited theoretical interests and possible applications for generalized exponential families, one research direction in information geometry is pointing to constructions of deformed geometries based on the standard one, keeping its basic properties. A typical and classical example of such a deformation would be the alpha-geometry [1,2], a statistical definition of which can be regarded as a replacement of the logarithmic function by suitable power functions. Hence, for the purpose of the generalization and flexible applicability, much attention is paid to various uses of such replacements by *representing functions* as important tools [3,4,11,12].

Two major characteristics of the standard structure are dual flatness and invariance [2]. Dual flatness (or Hessian structure [13]) produces fruitful properties such as the existence of canonical coordinate systems, a pair of conjugate potential functions and the canonical divergence (relative entropy). In addition, they are connected with the Legendre duality relation, which is also fundamental in the generalization of statistical physics. On the other hand, the invariance of geometric structure is crucially valuable in developing mathematical statistics. It has been proved [14] that invariance holds for only the structure with a special triple of a Riemannian metric and a pair of mutually dual affine connections, which are respectively called the Fisher information and the alpha-connections (see Section 3 for their definitions). The study of these two characteristics from a viewpoint of representing functions would contribute to our geometrical understanding.

In this paper, we first characterize a pair of representing functions that realizes the invariant information geometric structure. Next, we propose a new transformation to obtain dually flat geometries from a certain class of non-flat information geometries, using concepts from affine differential geometry [15,16]. We call the transformation *conformal flattening*, which is a generalization of the way to realize the corresponding dually flat geometry from the alpha-geometry developed in [17,18]. As applications and easy consequences of the results, we finally show several properties of gradient flows associated with realized dually flat geometries. Focusing on geometric characteristics conserved by the transformation, we discuss the properties such as a relation between geodesics and flows, the first integral of the flows and so on. These properties are new and general. Hence, they refine the arguments of the flows in [18], where only the alpha-geometry is treated.

The paper is organized as follows. In Section 2, we introduce preliminary results, explaining several existing methods to construct the information geometric structure that includes a dually flat structure and the alpha-structure and so on. We also give a short summary of concepts from affine differential geometry, which will be used in this paper. Section 3 provides a characterization of representing functions that realize invariant geometry, i.e., the one equipped with the Fisher information and a pair of the alpha-connections. The characterization is obtained by solving a simple system of ordinary equations. In Section 4, we first obtain a certain class of information geometric structure by regarding representing functions as immersions into an ambient affine space. Then, we demonstrate the conformal flattening to realize the corresponding dually flat structure, and discuss their properties and relations with generalized entropies or escort probabilities [19]. Section 5 exhibits the geometric properties of gradient flows with respect to a conformally realized Riemannian metric. These flows are reduced to the well-known *replicator flow* [20] (Chapter 16) when we consider the standard information geometry. Suitably choosing its pay-off functions, we see that the flow follows a geodesic curve or conserves a divergence from an equilibrium. In the final section, some concluding remarks are made.

Throughout the paper, we use a probability simplex as a statistical model for the sake of simplicity.

## 2. Preliminaries

### 2.1. Information Geometry of $S^{n}$ and $R_{+}^{n+1}$

Let us represent an element $p\in R^{n+1}$ with its components $p_{i},\;i=1,\cdots ,n+1$ as $p=\left(p_{i}\right)\in R^{n+1}$. Denote, respectively, the positive orthant by

$$R_{+}^{n+1}:=\left\{p=\left(p_{i}\right)\in R^{n+1}|p_{i}>0,\;i=1,\cdots ,n+1\right\},$$

and the relative interior of the probability simplex by

$$S^{n}:=\left\{p\in R_{+}^{n+1}\left|\sum_{i=1}^{n+1}p_{i}=1\right.\right\}.$$

Let p(X) be a probability distribution of a random variable *X* taking a value in the finite sample space Ω={1,2,⋯,n,n+1}. We consider a set of distributions p(X) with positive probabilities, i.e., $p\left(i\right)=p_{i}>0,\;i=1,\cdots ,n+1$, defined by

$$p\left(X\right)=\sum_{i=1}^{n+1}p_{i}\delta_{i}\left(X\right),\;\delta_{i}\left(j\right)=\delta_{i}^{j}\;\left(\mathrm{the}\;{\mathrm{Kronecker}}^{\prime }s\;\mathrm{delta}\right),$$

which is identified with $S^{n}$. A statistical model in $S^{n}$ is represented with parameters $\zeta =(\zeta^{j}),\;j=1,\cdots ,d\leq n$ by

$$p_{\zeta }\left(X\right)=\sum_{i=1}^{n+1}p_{i}\left(\zeta \right)\delta_{i}\left(X\right),$$

where each $p_{i}$ is smoothly parametrized by ζ. For such a statistical model, $\zeta^{j}$ can also be regarded as coordinates of the corresponding submanifold in $S^{n}$. For simplicity, we shall consider the full model, i.e., d=n and the parameter set is bijective with $S^{n}$ via $p_{i}\left(\zeta \right)$’s.

The information geometric structure [2] on $S^{n}$ denoted by $\left(g,\nabla ,\nabla^{*}\right)$ is composed of the pair of *mutually dual* torsion-free affine connections ∇ and $\nabla^{*}$ with respect to a Riemannian metric *g*. If we write $\partial_{i}:=\partial /\partial \zeta^{i},\;i=1,\cdots ,n$, the mutual duality requires components of $\left(g,\nabla ,\nabla^{*}\right)$ to satisfy

(1) $$\partial_{i}g_{jk}=\Gamma_{ij,k}+\Gamma_{ik,j}^{*}.$$

Let *L* and *M* be a pair of strictly monotone (i.e., one-to-one) smooth functions on the interval (0,1). One way of constructing such a structure $\left(g,\nabla ,\nabla^{*}\right)$ is to define the components as follows [2,11]:

(2) $$\begin{array}{ccc} g_{ij}\left(p\right) & = & \sum_{X\in \Omega }\partial_{i}L\left(p_{\zeta }\left(X\right)\right)\partial_{j}M\left(p_{\zeta }\left(X\right)\right),\;i,j=1,\cdots ,n, \end{array}$$

(3) $$\begin{array}{ccc} \Gamma_{ij,k}\left(p\right) & = & \sum_{X\in \Omega }\partial_{i}\partial_{j}L\left(p_{\zeta }\left(X\right)\right)\partial_{k}M\left(p_{\zeta }\left(X\right)\right),\;\;i,j,k=1,\cdots ,n, \end{array}$$

(4) $$\begin{array}{ccc} \Gamma_{ij,k}^{*}\left(p\right) & = & \sum_{X\in \Omega }\partial_{k}L\left(p_{\zeta }\left(X\right)\right)\partial_{i}\partial_{j}M\left(p_{\zeta }\left(X\right)\right),\;i,j,k=1,\cdots ,n. \end{array}$$

In this paper, we call *L* and *M* representing functions. It is easy to verify the mutual duality (1). (Positive definiteness of *g* needs additional conditions.)

When the curvature tensors of both ∇ and $\nabla^{*}$ vanish, $\left(g,\nabla ,\nabla^{*}\right)$ is called *dually flat* [2]. It is known that $\left(g,\nabla ,\nabla^{*}\right)$ is dually flat if and only if there exist two special coordinate systems denoted by $\theta^{i}\left(p\right)$ and $\eta_{i}\left(p\right),\;i=1,\cdots ,n$, respectively, where $\left(\theta^{i}\right)$ is ∇-affine, $\left(\eta_{i}\right)$ is $\nabla^{*}$-affine and they are biorthogonal, i.e.,

$$g\left(\frac{\partial }{\partial \theta^{i}},\frac{\partial }{\partial \eta_{j}}\right)=\delta_{i}^{j}.$$

We give examples. For a real number α, define $L^{\left(\alpha \right)}\left(u\right):=2u^{(1-\alpha )/2}/\left(1-\alpha \right)$ and $L^{\left(1\right)}:=\ln u$. If we set $L\left(u\right)=L^{\left(\alpha \right)}\left(u\right)$ and $M\left(u\right)=L^{\left(-\alpha \right)}\left(u\right)$, then they derive the *alpha-structure* [2] $\left(g^{F},\nabla^{\left(\alpha \right)},\nabla^{\left(-\alpha \right)}\right)$, where $g^{F}$ is the Fisher information and $\nabla^{\left(\pm \alpha \right)}$ are the alpha-connections (see Section 3). In particular, if we choose α=1, it defines the standard dually flat structure $\left(g^{F},\nabla^{\left(e\right)}:=\nabla^{\left(1\right)},\nabla^{\left(m\right)}:=\nabla^{\left(-1\right)}\right)$, where $\nabla^{\left(e\right)}$ and $\nabla^{\left(m\right)}$ are called the e- and m-connection, respectively [2]. Similarly, the ϕ-log geometry [3] can also be introduced in the same way by taking $L\left(u\right)=\log_{\phi }\left(u\right)$ and M(u)=u.

One traditional way to construct a general information geometric structure $\left(g,\nabla ,\nabla^{*}\right)$, without using representing functions, is by means of contrast functions (or divergences) [2,21]. In our case, let ρ be a function on $S^{n}\times S^{n}$ satisfying $\rho \left(p,r\right)\geq 0,\;\forall p,r\in S^{n}$ with equality if and only if p=r. For a vector field $\partial_{i}$, let ${\left(\partial_{i}\right)}_{p}$ denote its tangent vector at *p*. When we define

(5) $$\begin{array}{ccc} g_{ij}\left(p\right) & = & {\left.-{\left(\partial_{i}\right)}_{p}{\left(\partial_{j}\right)}_{r}\;\rho \left(p,r\right)\right|}_{p=r},\;\;i,j=1,\cdots ,n, \end{array}$$

(6) $$\begin{array}{ccc} \Gamma_{ij,k}\left(p\right) & = & {\left.-{\left(\partial_{i}\right)}_{p}{\left(\partial_{j}\right)}_{p}{\left(\partial_{k}\right)}_{r}\;\rho \left(p,r\right)\right|}_{p=r},\;\;i,j,k=1,\cdots ,n, \end{array}$$

(7) $$\begin{array}{ccc} \Gamma_{ij,k}^{*}\left(p\right) & = & {\left.-{\left(\partial_{i}\right)}_{p}{\left(\partial_{j}\right)}_{r}{\left(\partial_{k}\right)}_{r}\;\rho \left(p,r\right)\right|}_{p=r},\;\;i,j,k=1,\cdots ,n, \end{array}$$

we can confirm that (1) holds. If *g* is positive definite, we say that ρ is a *contrast function* or a *divergence* that induces the structure $\left(g,\nabla ,\nabla^{*}\right)$.

A contrast function ρ of the form:

(8) $$\rho \left(p,r\right)=\psi \left(\theta \left(p\right)\right)+\varphi \left(\eta \left(r\right)\right)-\sum_{i=1}^{n}\theta^{i}\left(p\right)\eta_{i}\left(r\right)$$

always induces the corresponding dually flat structure. Conversely, it is known [2] that if $\left(g,\nabla ,\nabla^{*}\right)$ is dually flat, then there exists the unique contrast function of the form (8) that induces the structure. Hence, it is called the *canonical divergence* of $\left(g,\nabla ,\nabla^{*}\right)$ and we say that the functions ψ and φ are *potentials*. By setting p=r, we see that a dually flat structure naturally gives the *Legendre duality* relations at each *p*, i.e., the function φ, is the Legendre conjugate of ψ satisfying

$$\eta_{i}=\frac{\partial \psi }{\partial \theta^{i}},\;\theta^{i}=\frac{\partial \varphi }{\partial \eta_{i}}.$$

Applying the idea of affine hypersurface theory [15] is also one of the other ways to construct the information geometric structure. Let *D* be the canonical flat affine connection on $R^{n+1}$. Consider an immersion *f* from $S^{n}$ into $R^{n+1}$ and a vector field ξ on $S^{n}$ that is transversal to the hypersurface $f(S^{n})$ in $R^{n+1}$. Such a pair (f,ξ), called an *affine immersion*, defines a torsion-free connection ∇ and the affine fundamental form *g* on $S^{n}$ via the Gauss formula as

(9) $$D_{X}f_{*}\left(Y\right)=f_{*}\left(\nabla_{X}Y\right)+g\left(X,Y\right)\xi ,\;X,Y\in X\left(S^{n}\right),$$

where $X(S^{n})$ is the set of tangent vector fields on $S^{n}$ and $f_{*}$ denotes the differential of *f*. By regarding *g* as a (pseudo-) Riemannian metric, one can discuss the realized structure (g,∇) on $S^{n}$.

We say that (f,ξ) is *non-degenerate* and *equiaffine* if *g* is non-degenerate and $D_{X}\xi$ is tangent to $S^{n}$ for any $X\in X(S^{n})$, respectively. The latter ensures that the volume element θ on $S^{n}$ defined by

$$\theta \left(X_{1},\cdots ,X_{n}\right)=\det\left(f_{*}\left(X_{1}\right),\cdots ,f_{*}\left(X_{n}\right),\xi \right),\;X_{i}\in X\left(S^{n}\right)$$

is parallel to ∇ [15] (p.31). It is known [15,16] that there exists a torsion-free dual affine connection $\nabla^{*}$ satisfying (1) if and only if (f,ξ) is non-degenerate and equiaffine. In this case, the obtained structure $\left(g,\nabla ,\nabla^{*}\right)$ on $S^{n}$ is not dually flat in general. However, there always exists a positive function σ and a dually flat structure $\left(\tilde{g},\tilde{\nabla },{\tilde{\nabla }}^{*}\right)$ on $S^{n}$ that hold the following relations [16]:

(10) $$\begin{array}{ccc} \tilde{g} & = & \sigma g, \end{array}$$

(11) $$\begin{array}{ccc} g({\tilde{\nabla }}_{X}Y,Z) & = & g\left(\nabla_{X}Y,Z\right)-d\left(\ln\sigma \right)\left(Z\right)g\left(X,Y\right), \end{array}$$

(12) $$\begin{array}{ccc} g({\tilde{\nabla }}_{X}^{*}Y,Z) & = & g\left(\nabla_{X}^{*}Y,Z\right)+d\left(\ln\sigma \right)\left(X\right)g\left(Y,Z\right)+d\left(\ln\sigma \right)\left(Y\right)g\left(X,Z\right). \end{array}$$

Furthermore, there exists a specific contrast function ρ(p,r) for $\left(g,\nabla ,\nabla^{*}\right)$ called the *geometric divergence*. Then, a contrast function $\tilde{\rho }\left(p,r\right)$ that induces $\left(\tilde{g},\tilde{\nabla },{\tilde{\nabla }}^{*}\right)$ is given by the *conformal divergence* $\tilde{\rho }\left(p,r\right)=\sigma \left(r\right)\rho \left(p,r\right)$. These properties of the structure (g,∇) realized by the non-degenerate and equiaffine immersion are called *1-conformal flatness* [16].

## 3. Characterization of Invariant Geometry by Representing Functions

Suppose that a pair of representing functions (L,M) defines an information geometric structure $\left(g,\nabla ,\nabla^{*}\right)$ by (2), (3) and (4). In this section, we consider the condition of (L,M) such that $\left(g,\nabla ,\nabla^{*}\right)$ is invariant. This is equivalent [2,14] to *g* which is the Fisher information $g^{F}$ defined by

(13) $$g_{ij}^{F}\left(p\right)=\sum_{X\in \Omega }p_{\zeta }\left(\partial_{i}\ln p_{\zeta }\right)\left(\partial_{j}\ln p_{\zeta }\right)$$

and a pair of dual connections satisfies $\nabla =\nabla^{\left(\alpha \right)}$ and $\nabla^{*}=\nabla^{\left(-\alpha \right)}$ for a certain α∈R, where $\nabla^{\left(\alpha \right)}$ is the α-connection defined by

(14) $$\Gamma_{ij,k}^{\left(\alpha \right)}=\sum_{X\in \Omega }p_{\zeta }\left(\partial_{i}\partial_{j}\ln p_{\zeta }+\frac{1-\alpha }{2}\left(\partial_{i}\ln p_{\zeta }\right)\left(\partial_{j}\ln p_{\zeta }\right)\right)\left(\partial_{k}\ln p_{\zeta }\right).$$

Hence, $g_{ij}$ expressed in (2) by functions L(u) and M(u) coincides with the Fisher information if and only if the following equation holds:

(15) $$\frac{dL}{du}\frac{dM}{du}=1/u.$$

Similarly, we derive a condition for $\Gamma_{ij,k}$ expressed in (3) to be the α-connection. First, note that the following relations hold:

(16) $$\partial_{i}\ln p_{\zeta }=\left(\partial_{i}p_{\zeta }\right)\frac{1}{p_{\zeta }},\;\partial_{i}\partial_{j}\ln p_{\zeta }=\left(\partial_{i}\partial_{j}p_{\zeta }\right)\frac{1}{p_{\zeta }}-\left(\partial_{i}p_{\zeta }\right)\left(\partial_{j}p_{\zeta }\right)\frac{1}{p_{\zeta }^{2}}.$$

On the other hand, we have

(17) $$\partial_{i}\partial_{j}L\left(p_{\zeta }\right)=\left(\partial_{i}\partial_{j}p_{\zeta }\right)\frac{dL}{du}\left(p_{\zeta }\right)+\left(\partial_{i}p_{\zeta }\right)\left(\partial_{j}p_{\zeta }\right)\frac{d^{2}L}{du^{2}}\left(p_{\zeta }\right),$$

(18) $$\partial_{k}M\left(p_{\zeta }\right)=\left(\partial_{k}p_{\zeta }\right)\frac{dM}{du}\left(p_{\zeta }\right).$$

Substituting (16), (17) and (18) into (3) and (14), and comparing them, we obtain (15) again and

(19) $$\frac{d^{2}L}{du^{2}}\frac{dM}{du}=-\frac{1+\alpha }{2u^{2}}.$$

Expressing $L^{\prime }:=dL/du$ and $L^{\prime \prime }:=d^{2}L/du^{2}$, we have the following ODE from (15) and (19):

(20) $$\frac{L^{\prime \prime }}{L^{\prime }}=-\frac{1+\alpha }{2u}.$$

By integrations, we get

(21) $$\ln L^{\prime }=-\frac{\left(1+\alpha \right)}{2}\ln u+c$$

and

(22) $$L\left(u\right)=c_{1}u^{(1-\alpha )/2}+c_{2},\;M\left(u\right)=c_{3}u^{(1+\alpha )/2}+c_{4},$$

where *c* and $c_{i},\;i=1,\cdots ,4$ are constants with a constraint $c_{1}c_{3}=4/\left(1-\alpha^{2}\right)$. Thus, (L,M) is essentially a pair of representing functions that derives the alpha-geometry and there is only freedom of adjusting the constants for the invariance of geometry. If we require solely (15), which implies that only a Riemannian metric *g* is the Fisher information $g^{F}$, there still remains much freedom for (L,M).

## 4. Affine Immersion of the Probability Simplex

Now we consider the affine immersion with the following assumptions.

**Assumptions:**

1. The affine immersion (f,ξ) is nondegenerate and equiaffine,
2. The immersion *f* is given by the component-by-component and common representing function *L*, i.e.,
  $$f:S^{n}∋p=\left(p_{i}\right)\mapsto x=\left(x^{i}\right)\in R^{n+1},\;x^{i}=L\left(p_{i}\right),\;i=1,\cdots ,n+1,$$
3. The representing function L:(0,1)→R is sign-definite, concave with $L^{\prime \prime }<0$ and strictly increasing, i.e., $L^{\prime }>0$. Hence, the inverse of *L* denoted by *E* exists, i.e., E∘L=id.
4. Each component of ξ satisfies $\xi^{i}<0,\;i=1,\cdots ,n+1$ on $S^{n}$.

**Remark** **1.** *From the assumption 3, it follows that $L^{\prime }E^{\prime }=1$, $E^{\prime }>0$ and $E^{\prime \prime }>0$. Regarding sign-definiteness of L, note that we can adjust L(u) to L(u)+c by a suitable constant c without loss of generality since the resultant geometric structure is unchanged (See Theorem 1) by the adjustment. For a fixed L satisfying the assumption 3, we can choose ξ that meets the assumptions 1 and 4. For example, if we take $\xi^{i}=-\left|L\left(p_{i}\right)\right|$, then (f,ξ) is called *centro-affine*, which is known to be equiaffine [15] (p.37). The assumptions 3 and 4 also assure positive definiteness of g (the details are described in the proof of Theorem 1). Hence, (f,ξ) is non-degenerate and we can regard g as a Riemannian metric on $S^{n}$.*

### 4.1. Conormal Vector and the Geometric Divergence

Define a function Ψ on $R^{n+1}$ by

$$\Psi \left(x\right):=\sum_{i=1}^{n+1}E\left(x^{i}\right),$$

then $f(S^{n})$ immersed in $R^{n+1}$ is expressed as a level surface of Ψ(x)=1. Denote by $R_{n+1}$ the dual space of $R^{n+1}$ and by 〈ν,x〉 the pairing of $x\in R^{n+1}$ and $\nu \in R_{n+1}$. The conormal vector [15] (p.57) $\nu :S^{n}\to R_{n+1}$ for the affine immersion (f,ξ) is defined by

(23) $$\langle \nu \left(p\right),f_{*}\left(X\right)\rangle =0,\;\forall X\in T_{p}S^{n},\;\langle \nu \left(p\right),\xi \left(p\right)\rangle =1,$$

for $p\in S^{n}$. Using the assumptions and noting the relations:

$$\frac{\partial \Psi }{\partial x^{i}}=E^{\prime }\left(x^{i}\right)=\frac{1}{L^{\prime }\left(p_{i}\right)}>0,\;i=1,\cdots ,n+1,$$

we have

(24) $$\nu_{i}\left(p\right):=\frac{1}{\Lambda }\frac{\partial \Psi }{\partial x^{i}}=\frac{1}{\Lambda (p)}E^{\prime }\left(x^{i}\right)=\frac{1}{\Lambda (p)}\frac{1}{L^{\prime }\left(p_{i}\right)},\;i=1,\cdots ,n+1,$$

where Λ is a normalizing factor defined by

(25) $$\Lambda \left(p\right):=\sum_{i=1}^{n+1}\frac{\partial \Psi }{\partial x^{i}}\xi^{i}=\sum_{i=1}^{n+1}\frac{1}{L^{\prime }\left(p_{i}\right)}\xi^{i}\left(p\right).$$

Then, we can confirm (23) using the relation $\sum_{i=1}^{n+1}X^{i}=0$ for $X=\left(X^{i}\right)\in X\left(S^{n}\right)$. Note that $v:S^{n}\to R_{n+1}$ defined by

$$v_{i}\left(p\right)=\Lambda \left(p\right)\nu_{i}\left(p\right)=\frac{1}{L^{\prime }\left(p_{i}\right)},\;i=1,\cdots ,n+1,$$

also satisfies

(26) $$\begin{array}{c} \langle v\left(p\right),f_{*}\left(X\right)\rangle =0,\;\forall X\in T_{p}S^{n}. \end{array}$$

Furthermore, it follows, from (24), (25) and the assumption 4, that

$$\Lambda \left(p\right)<0,\;\nu_{i}\left(p\right)<0,\;i=1,\cdots ,n+1,$$

for all $p\in S^{n}$.

It is known [15] (p.57) that the affine fundamental form *g* can be represented by

$$g\left(X,Y\right)=-\langle \nu_{*}\left(X\right),f_{*}\left(Y\right)\rangle ,\;X,Y\in T_{p}S^{n}.$$

In our case, it is calculated via (26) as

(27) $$\begin{array}{ccc} g(X,Y) & = & -\Lambda^{-1}\langle v_{*}\left(X\right),f_{*}\left(Y\right)\rangle -X\left(\Lambda^{-1}\right)\langle v,f_{*}\left(Y\right)\rangle \\ & = & -\frac{1}{\Lambda }\sum_{i=1}^{n+1}{\left(\frac{1}{L^{\prime }\left(p_{i}\right)}\right)}^{\prime }L^{\prime }\left(p_{i}\right)X^{i}Y^{i}=\frac{1}{\Lambda }\sum_{i=1}^{n+1}\frac{L^{\prime \prime }\left(p_{i}\right)}{L^{\prime }\left(p_{i}\right)}X^{i}Y^{i},\;X,Y\in T_{p}S^{n}. \end{array}$$

Hence, *g* is positive definite from the assumptions 3 and 4, and we can regard it as a Riemannian metric.

Utilizing these notions from affine differential geometry, we can introduce a geometric divergence [16] as follows:

(28) $$\begin{array}{ccc} \rho (p,r) & = & \langle \nu \left(r\right),f\left(p\right)-f\left(r\right)\rangle =\sum_{i=1}^{n+1}\nu_{i}\left(r\right)\left(L\left(p_{i}\right)-L\left(r_{i}\right)\right)\\ & = & \frac{1}{\Lambda (r)}\sum_{i=1}^{n+1}\frac{L\left(p_{i}\right)-L\left(r_{i}\right)}{L^{\prime }\left(r_{i}\right)},\;p,r\in S^{n}. \end{array}$$

It is easily checked that ρ is actually a contrast function of the 1-conformally flat structure $\left(g,\nabla ,\nabla^{*}\right)$ using (5), (6) and (7).

### 4.2. Conformal Flattening Transformation

As is described in the preliminary section, by 1-conformally flatness there exists a positive function, i.e., conformal factor σ that relates $\left(g,\nabla ,\nabla^{*}\right)$ with a dually flat structure $\left(\tilde{g},\tilde{\nabla },{\tilde{\nabla }}^{*}\right)$ via the conformal transformation (10), (11) and (12). A contrast function $\tilde{\rho }$ that induces $\left(\tilde{g},\tilde{\nabla },{\tilde{\nabla }}^{*}\right)$ is given as the conformal divergence:

(29) $$\tilde{\rho }\left(p,r\right)=\sigma \left(r\right)\rho \left(p,r\right),\;p,r\in S^{n}.$$

from the geometric divergence ρ in (28).

For an arbitrary function *L* within our setting given by the four assumptions, we prove that we can construct a dually flat structure $\left(\tilde{g},\tilde{\nabla },{\tilde{\nabla }}^{*}\right)$ by choosing the conformal factor σ carefully. Hereafter, we call this transformation *conformal flattening*.

Define

$$Z\left(p\right):=\sum_{i=1}^{n+1}\nu_{i}\left(p\right)=\frac{1}{\Lambda (p)}\sum_{i=1}^{n+1}\frac{1}{L^{\prime }\left(p_{i}\right)},$$

then it is negative because each $\nu_{i}\left(p\right)$ is negative. The conformal divergence to ρ with respect to the conformal factor σ(r):=−1/Z(r) is

$$\tilde{\rho }\left(p,r\right)=-\frac{1}{Z(r)}\rho \left(p,r\right).$$

**Theorem** **1.** If the conformal factor is σ=−1/Z, then the information geometric structure $\left(\tilde{g},\tilde{\nabla },{\tilde{\nabla }}^{*}\right)$ on $S^{n}$ that is transformed from the 1-conformally flat structure $\left(g,\nabla ,\nabla^{*}\right)$ via (10), (11) and (12) is dully flat. Furthermore, the conformal divergence $\tilde{\rho }$ that induces $\left(\tilde{g},\tilde{\nabla },{\tilde{\nabla }}^{*}\right)$ on $S^{n}$ is canonical where Legendre conjugate potential functions and coordinate systems are explicitly given by

(30) $$\begin{array}{ccc} \theta^{i}\left(p\right) & = & x^{i}\left(p\right)-x^{n+1}\left(p\right)=L\left(p_{i}\right)-L\left(p_{n+1}\right),\;i=1,\cdots ,n, \end{array}$$

(31) $$\begin{array}{ccc} \eta_{i}\left(p\right) & = & P_{i}\left(p\right):=\frac{\nu_{i}\left(p\right)}{Z(p)}=\frac{1/L^{\prime }\left(p_{i}\right)}{\sum_{k=1}^{n+1}1/L^{\prime }\left(p_{k}\right)},\;i=1,\cdots ,n, \end{array}$$

(32) $$\begin{array}{ccc} \psi (p) & = & -x_{n+1}\left(p\right)=-L\left(p_{n+1}\right), \end{array}$$

(33) $$\begin{array}{ccc} \varphi (p) & = & \frac{1}{Z(p)}\sum_{i=1}^{n+1}\nu_{i}\left(p\right)x^{i}\left(p\right)=\sum_{i=1}^{n+1}P_{i}\left(p\right)L\left(p_{i}\right). \end{array}$$

**Proof.**  Using given relations, we first show that the conformal divergence $\tilde{\rho }$ is the canonical divergence for $\left(\tilde{g},\tilde{\nabla },{\tilde{\nabla }}^{*}\right)$:

(34) $$\begin{array}{ccc} \tilde{\rho }\left(p,r\right) & = & -\frac{1}{Z(r)}\langle \nu \left(r\right),f\left(p\right)-f\left(r\right)\rangle =\langle P\left(r\right),f\left(r\right)-f\left(p\right)\rangle \\ & = & \sum_{i=1}^{n+1}P_{i}\left(r\right)\left(x^{i}\left(r\right)-x^{i}\left(p\right)\right)\\ & = & \sum_{i=1}^{n+1}P_{i}\left(r\right)x^{i}\left(r\right)-\sum_{i=1}^{n}P_{i}\left(r\right)\left(x^{i}\left(p\right)-x^{n+1}\left(p\right)\right)-\left(\sum_{i=1}^{n+1}P_{i}\left(r\right)\right)x^{n+1}\left(p\right)\\ & = & \varphi \left(r\right)-\sum_{i=1}^{n}\eta_{i}\left(r\right)\theta^{i}\left(p\right)+\psi \left(p\right). \end{array}$$

Next, let us confirm that $\partial \psi /\partial \theta^{i}=\eta_{i}$.

Since $\theta^{i}\left(p\right)=L\left(p_{i}\right)+\psi \left(p\right),\;i=1,\cdots ,n$, we have

$$p_{i}=E\left(\theta^{i}-\psi \right),\;i=1,\cdots ,n+1,$$

by setting $\theta^{n+1}:=0$. Hence, we have

$$1=\sum_{i=1}^{n+1}E\left(\theta^{i}-\psi \right).$$

Differentiating by $\theta^{j}$, we obtain

$$\begin{array}{ccc} 0 & = & \frac{\partial }{\partial \theta^{j}}\sum_{i=1}^{n+1}E\left(\theta^{i}-\psi \right)=\sum_{i=1}^{n+1}E^{\prime }\left(\theta^{i}-\psi \right)\left(\delta_{j}^{i}-\frac{\partial \psi }{\partial \theta^{j}}\right)\\ & = & E^{\prime }\left(x^{j}\right)-\left(\sum_{i=1}^{n+1}E^{\prime }\left(x^{i}\right)\right)\frac{\partial \psi }{\partial \theta^{j}}. \end{array}$$

This implies that

$$\frac{\partial \psi }{\partial \theta^{j}}=\frac{E^{\prime }\left(x^{j}\right)}{\sum_{i=1}^{n+1}E^{\prime }\left(x^{i}\right)}=\eta_{j}.$$

Together with (34) and this relation, φ is confirmed to be the Legendre conjugate of ψ.

The dual relation $\partial \varphi /\partial \eta_{i}=\theta^{i}$ follows automatically from the property of the Legendre transform. □

The following corollary is straightforward because all the quantities in the theorem depend on only *L*:

**Corollary** **1.** Under the assumptions, the dually flat structure $\left(\tilde{g},\tilde{\nabla },{\tilde{\nabla }}^{*}\right)$ on $S^{n}$, obtained by following the above conformal flattening, does not depend on the choice of the transversal vector ξ.

**Remark** **2.** Note that the conformal metric is given by $\tilde{g}=-g/Z$ and is positive definite. Furthermore, the relation (12) means that the dual affine connections $\nabla^{*}$ and ${\tilde{\nabla }}^{*}$ are projectively* (or* -1-conformally*)* equivalent [15,16]. Hence, $\nabla^{*}$ is projectively flat. Furthermore, the above corollary implies that the realized affine connection ∇ *is also projectively equivalent to the flat connection $\tilde{\nabla }$ if we use the centro-affine immersion, i.e., $\xi^{i}=-L\left(p_{i}\right)$ [15,16]. See Proposition 3 for an application of projective equivalence of affine connections.*

**Remark** **3.** *In our setting, conformal flattening is geometrically regarded as normalization of the conormal vector ν. Hence, the dual coordinates $\eta_{i}\left(p\right)=P_{i}\left(p\right)$ can be interpreted as a generalization of the* escort probability* [10,19] (see the following example). Similarly, ψ and −φ might be seen as the associated Massieu function and entropy, respectively.*

**Remark** **4.** While the immersion f is composed of a representing function L under the assumption 2, the corresponding M of a single variable does not generally exist for $\left(g,\nabla ,\nabla^{*}\right)$ nor $\left(\tilde{g},\tilde{\nabla },{\tilde{\nabla }}^{*}\right)$. From the expressions of the Riemann metrics g in (27) and $\tilde{g}=-g/Z$, we see that the counterparts of the representing functions $M(p_{i})$ would be, respectively, $-\nu_{i}\left(p\right)$ and $P_{i}\left(p\right)$, but note that they are multi-variable functions of $p=(p_{i})$.

### 4.3. Examples

If we take *L* to be the logarithmic function L(t)=ln(t), then the conformally flattened geometry immediately defines the standard dually flat structure $\left(g^{F},\nabla^{\left(1\right)},\nabla^{\left(-1\right)}\right)$ on the simplex $S^{n}$. We see that −φ(p) is the entropy, i.e., $\varphi \left(p\right)=\sum_{i=1}^{n+1}p_{i}\ln p_{i}$ and the conformal divergence is the KL divergence (relative entropy), i.e., $\tilde{\rho }\left(p,r\right)=D^{\left(\mathrm{KL}\right)}\left(r||p\right)=\sum_{i=1}^{n+1}r_{i}\left(\ln r_{i}-\ln p_{i}\right)$.

Next, let the affine immersion (f,ξ) be defined by the following *L* and ξ:

$$L\left(t\right):=\frac{1}{1-q}t^{1-q},\;x^{i}\left(p\right)=\frac{1}{1-q}{\left(p_{i}\right)}^{1-q},$$

and

$$\xi^{i}\left(p\right)=-q\left(1-q\right)x^{i}\left(p\right),$$

with 0<q and q≠1. We see that the immersion is centro-affine scaled by the constant factor q(1−q). Then, we see that the immersion realizes the alpha-structure $\left(g^{F},\nabla^{\left(\alpha \right)},\nabla^{\left(-\alpha \right)}\right)$ on $S^{n}$ with q=(1+α)/2. The geometric divergence is the alpha-divergence, i.e.,

$$\rho \left(p,r\right)=\frac{4}{1-\alpha^{2}}\left(1-\sum_{i=1}^{n+1}{\left(p_{i}\right)}^{(1-\alpha )/2}{\left(r_{i}\right)}^{(1+\alpha )/2}\right).$$

Following the procedure of conformal flattening described in the above, we have [17]

$$\Psi \left(x\right)=\sum_{i=1}^{n+1}{\left(\left(1-q\right)x^{i}\right)}^{1/1-q},\;\Lambda \left(p\right)=-q,\;\left(constant\right)$$

$$\nu_{i}\left(p\right)=-\frac{1}{q}{\left(p_{i}\right)}^{q},\;\sigma \left(p\right)=-\frac{1}{Z(p)}=\frac{q}{\sum_{k=1}^{n+1}{\left(p_{i}\right)}^{q}},$$

and obtain a dually flat structure $\left({\tilde{g}}^{F},\tilde{\nabla },{\tilde{\nabla }}^{*}\right)$ via the formulas in Theorem 1:

$$\eta_{i}=P_{i}=\frac{{\left(p_{i}\right)}^{q}}{\sum_{k=1}^{n+1}{\left(p_{k}\right)}^{q}},\;\theta^{i}=\frac{1}{1-q}{\left(p_{i}\right)}^{1-q}-\frac{1}{1-q}{\left(p_{n+1}\right)}^{1-q}=\ln_{q}\left(p_{i}\right)-\psi \left(p\right),$$

$$\psi \left(p\right)=-\ln_{q}\left(p_{n+1}\right),\;\varphi \left(p\right)=\ln_{q}\left(\frac{1}{\exp_{q}\left(S_{q}\left(p\right)\right)}\right),\;{\tilde{g}}^{F}=-\frac{1}{Z(p)}g^{F}.$$

Here, $\ln_{q}$ and $S_{q}\left(p\right)$ are the *q-logarithmic function* and the *Tsallis entropy* [10], respectively, defined by

$$\ln_{q}\left(t\right)=\frac{t^{1-q}-1}{1-q},\;S_{q}\left(p\right)=\frac{\sum_{i=1}^{n+1}{\left(p_{i}\right)}^{q}-1}{1-q}.$$

Note that the escort probability appears as the dual coordinate $\eta_{i}$.

## 5. An Application to Gradient Flows on $S^{n}$

Recall the *replicator flow* on the simplex $S^{n}$ for given functions $f_{i}\left(p\right)$ defined by

(35) $${\dot{p}}_{i}=p_{i}\left(f_{i}\left(p\right)-\bar{f}\left(p\right)\right),\;i=1,\cdots ,n+1,\;\bar{f}\left(p\right):=\sum_{i=1}^{n+1}p_{i}f_{i}\left(p\right),$$

which is extensively studied in evolutionary game theory. It is known [20] (Chapter 16) that

(i) the solution to (35) is the gradient flow that maximizes a function V(p) satisfying
  (36) $$f_{i}=\frac{\partial V}{\partial p_{i}},\;i=1,\cdots ,n+1,$$
  with respect to the *Shahshahani metric* $g^{S}$ (See below),
(ii) the KL divergence is a local Lyapunov function for an equilibrium called the *evolutionary stable state (ESS)* for the case of $f_{i}\left(p\right)=\sum_{j=1}^{n+1}a_{ij}p_{j}$ with $\left(a_{ij}\right)\in R^{\left(n+1)\times (n+1\right)}$.

The Shahshahani metric $g^{S}$ is defined on the positive orthant $R_{+}^{n+1}$ by

$$g_{ij}^{S}\left(p\right)=\frac{\sum_{k=1}^{n+1}p_{k}}{p_{i}}\delta_{ij},\;i,j=1,\cdots ,n+1.$$

Note that the Shahshahani metric induces the Fisher metric $g^{F}$ on $S^{n}$. Further, the KL divergence is the canonical divergence [2] of $\left(g^{F},\nabla^{\left(1\right)},\nabla^{\left(-1\right)}\right)$. Thus, the replicator dynamics (35) are closely related with the standard dually flat structure $\left(g^{F},\nabla^{\left(1\right)},\nabla^{\left(-1\right)}\right)$, which associates with exponential and mixture families of probability distributions. In addition, investigation of the flow is also important from a viewpoint of statistical physics governed by the Boltzmann–Gibbs distributions when we choose V(p) as various physical quantities, e.g., free energy or entropy.

Similarly, when we consider various Legendre relations deformed by *L*, it would be of interest to investigate gradient flows on $S^{n}$ for a dually flat structure $\left(\tilde{g},\tilde{\nabla },{\tilde{\nabla }}^{*}\right)$ or a 1-conformally flat structure $\left(g,\nabla ,\nabla^{*}\right)$. Since *g* and $\tilde{g}$ can be naturally extended to $R_{+}^{n+1}$ as a diagonal form (we use the same notation for brevity):

(37) $$g_{ij}\left(p\right)=\frac{1}{\Lambda (p)}\frac{L^{\prime \prime }\left(p_{i}\right)}{L^{\prime }\left(p_{i}\right)}\delta_{ij},\;{\tilde{g}}_{ij}\left(p\right)=-\frac{1}{Z(p)}g_{ij}\left(p\right),\;i,j=1,\cdots ,n+1$$

from (27), we can define two gradient flows for V(p) on $S^{n}$. One is the gradient flow for *g*, which is

(38) $${\dot{p}}_{i}=g_{ii}^{-1}\left(f_{i}-{\bar{f}}^{H}\right),\;{\bar{f}}^{H}\left(p\right):=\sum_{k=1}^{n+1}H_{k}\left(p\right)f_{k}\left(p\right),\;H_{i}\left(p\right):=\frac{g_{ii}^{-1}\left(p\right)}{\sum_{k=1}^{n+1}g_{kk}^{-1}\left(p\right)},$$

for i=1,⋯,n+1. It is verified that $\dot{p}$ is tangent to $S^{n}$, i.e., $\dot{p}\in T_{p}S^{n}$ and gradient of *V*, i.e.,

$$g\left(X,\dot{p}\right)=\sum_{i=1}^{n+1}f_{i}X^{i}-{\bar{f}}^{H}\sum_{i=1}^{n+1}X^{i}=\sum_{i=1}^{n+1}\frac{\partial V}{\partial p_{i}}X^{i},\;\forall X=\left(X^{i}\right)\in X\left(S^{n}\right).$$

In the same way, the other one for $\tilde{g}$ is defined by

(39) $${\dot{p}}_{i}={\tilde{g}}_{ii}^{-1}\left(f_{i}-{\bar{f}}^{H}\right),\;{\bar{f}}^{H}\left(p\right):=\sum_{k=1}^{n+1}H_{k}\left(p\right)f_{k}\left(p\right),\;i=1,\cdots ,n+1.$$

Note that both the flows reduce to (35) when L=ln.

From (37), the following consequence is immediate:

**Proposition** **1.** The trajectories of the gradient flow (38) and (39) starting from the same initial point coincide while velocities of time-evolutions are different by the factor-Z(p).

Taking account of the example with respect to the alpha-geometry and the conformally flattened one given in subsection 4.3, the following result shown in [18] can be regarded as a corollary of the above proposition:

**Corollary** **2.** The trajectories of the gradient flow (39) with respect to the conformal metric $\tilde{g}$ for $L\left(t\right)=t^{1-q}/\left(1-q\right)$ coincide with those of the replicator flow (35) while velocities of time-evolutions are different by the factor-Z(p).

Next, we particularly consider the case when V(p) is a potential function or divergences. As for a gradient flow on a manifold equipped with a dually flat structure $\left(\tilde{g},\tilde{\nabla },{\tilde{\nabla }}^{*}\right)$, the following result is known:

**Proposition** **2.** [22] Consider the potential function ψ(p) and the canonical divergence $\tilde{\rho }\left(p,r\right)$ of $\left(\tilde{g},\tilde{\nabla },{\tilde{\nabla }}^{*}\right)$ for an arbitrary prefixed point r. The gradient flows for V(p)=±ψ(p) and V(p)=±ρ(p,r) follow ${\tilde{\nabla }}^{*}$-geodesic curves.

As is described in Remark 2, $\nabla^{*}$ and ${\tilde{\nabla }}^{*}$ are *projectively equivalent*. One geometrically interesting property of the projective equivalence is that $\nabla^{*}$- and ${\tilde{\nabla }}^{*}$- geodesic curves coincide up to their parametrizations (i.e., a curve is $\nabla^{*}$-pregeodesic if and only if it is ${\tilde{\nabla }}^{*}$-pregeodesic) [15] (p.17). Combining this fact with Propositions 1 and 2, we see that the following result holds:

**Proposition** **3.** Let $r\in S^{n}$ be an arbitrary prefixed point. The gradient flows (38) for $V\left(p\right)=\pm \rho \left(p,r\right)=\pm \tilde{\rho }\left(p,r\right)/\sigma \left(r\right)$, $V\left(p\right)=\pm \tilde{\rho }\left(p,r\right)$ and V(p)=±ψ(p) follow ${\tilde{\nabla }}^{*}$-geodesic curves.

Finally, we demonstrate here another aspect of the flow (39). Let us particularly consider the following functions $f_{i}$:

(40) $$f_{i}\left(p\right):=\frac{L^{\prime \prime }\left(p_{i}\right)}{{\left(L^{\prime }\left(p_{i}\right)\right)}^{2}}\sum_{j=1}^{n+1}a_{ij}P_{j}\left(p\right),\;a_{ij}=-a_{ji}\in R,\;i,j=1,\cdots ,n+1.$$

Note that $f_{i}$s are not integrable, i.e., non-trivial *V* satisfying (36) does not exist because of the anti-symmetry of $a_{ij}$. Hence, for this case, (39) is no longer a gradient flow. However, we can prove the following result:

**Theorem** **2.** Consider the flow (39) with the functions $f_{i}$s defined in (40) and assume that there exists an equilibrium $r\in S^{n}$ for the flow. Then, ρ(p,r) and $\tilde{\rho }\left(p,r\right)$ are the first integral (conserved quantity) of the flow.

**Proof.**  By substituting (40) into ${\bar{f}}^{H}\left(p\right)$ in (39) and using the expression of ${\tilde{g}}_{ii}$ in (37), we have

$$\begin{array}{c} {\bar{f}}^{H}\left(p\right)=\frac{1}{\sum_{k=1}^{n+1}L^{\prime }\left(p_{k}\right)/L^{\prime \prime }\left(p_{k}\right)}\sum_{i=1}^{n+1}\frac{1}{L^{\prime }\left(p_{i}\right)}\sum_{j=1}^{n+1}a_{ij}P_{i}\left(p\right). \end{array}$$

By the relation $E^{\prime }\left(x_{i}\right)=1/L^{\prime }\left(p_{i}\right)$ and (31), it holds that

$$\sum_{i=1}^{n+1}\frac{1}{L^{\prime }\left(p_{i}\right)}\sum_{j=1}^{n+1}a_{ij}P_{i}\left(p\right)=\frac{1}{\sum_{k=1}^{n+1}E^{\prime }\left(x_{k}\right)}\sum_{i=1}^{n+1}\sum_{j=1}^{n+1}a_{ij}E^{\prime }\left(x_{l}\right)E^{\prime }\left(x_{j}\right)=0.$$

Hence, we see that ${\bar{f}}^{H}=0$ and the flow (39) reduces to

(41) $${\dot{p}}_{i}={\tilde{g}}_{ii}^{-1}\left(p\right)f_{i}\left(p\right),\;i=1,\cdots ,n+1.$$

Since *r* is an equilibrium point, we see from (40) that

(42) $$\sum_{j=1}^{n+1}a_{ij}P_{j}\left(r\right)=0,\;i=1,\cdots ,n+1.$$

Then, using (34), (41) and (42), we have

$$\begin{array}{ccc} \frac{d\tilde{\rho }\left(p,r\right)}{dt} & = & -\sum_{i=1}^{n+1}P_{i}\left(r\right)L^{\prime }\left(p_{i}\right){\dot{p}}_{i}\\ & = & Z\left(p\right)\Lambda \left(p\right)\sum_{i=1}^{n+1}P_{i}\left(r\right)\frac{{\left(L^{\prime }\left(p_{i}\right)\right)}^{2}}{L^{\prime \prime }\left(p_{i}\right)}f_{i}\left(p\right)=Z\left(p\right)\Lambda \left(p\right)\sum_{i=1}^{n+1}P_{i}\left(r\right)\left(\sum_{j=1}^{n+1}a_{ij}P_{j}\left(p\right)\right)\\ & = & Z\left(p\right)\Lambda \left(p\right)\sum_{i=1}^{n+1}\sum_{j=1}^{n+1}\left(P_{i}\left(p\right)-P_{i}\left(r\right)\right)a_{ij}P_{j}\left(p\right)\\ & = & Z\left(p\right)\Lambda \left(p\right)\sum_{i=1}^{n+1}\sum_{j=1}^{n+1}\left(P_{i}\left(p\right)-P_{i}\left(r\right)\right)a_{ij}\left(P_{j}\left(p\right)-P_{j}\left(r\right)\right)=0. \end{array}$$

Thus, $\tilde{\rho }\left(p,r\right)$ is the first integral of the flow. It follows that ρ(p,r) is also the first integral of the flow from the definition of conformal divergence (29). □

**Remark** **5.** From proposition 1, the same statement holds for the flow (38). The proposition implies the fact [20] that the KL divergence is the first integral for the replicator flow (35) with the function $f_{i}\left(p\right)$ in (40) defined by L(t)=lnt and $P_{j}\left(p\right)=p_{j}$.

## 6. Conclusions

We have considered two important aspects of information geometric structure, i.e., invariance and dual flatness, from a viewpoint of representing functions. As for the invariance of geometry, we have proved that a pair of representing functions that derives the alpha-structure is essentially unique. On the other hand, we have shown the explicit formula of conformal flattening that transforms 1-conformally flat structures on the simplex $S^{n}$ realized by affine immersions to the corresponding dually flat structures. Finally, we have discussed several geometric properties of gradient flows associated to two structures.

Presently, our analysis is restricted to the probability simplex, i.e., the space of discrete probability distributions. For the continuous case, the similar or related results are obtained in [23,24] without using affine immersions. Extensions of the results obtained in this paper to continuous probability space and the exploitation of relations to the literature are left for future work.

The conformal flattening can also be applied to the computationally efficient construction of a Voronoi diagram with respect to the geometric divergences [18]. Exploring the possibilities of other applications would be of interest.
